# Sensory processing and its relevance to occupational performance: a systematic review

**DOI:** 10.3389/fpsyg.2026.1844400

**Published:** 2026-05-25

**Authors:** Alicia Gibello-Rufo, Elisa M. Garrido-Ardila, María Jiménez-Palomares, María Carmen Cilleros-Sánchez, Blanca González-Sánchez, Juan Rodríguez-Mansilla

**Affiliations:** 1 Faculty of Nursing and Occupational Therapy at the University of Extremadura, Cáceres, Spain; 2 ADOLOR Research Group, Department of Medical-Surgical Therapy, Medicine Faculty Extremadura University, Badajoz, Spain

**Keywords:** activities of daily living, occupational therapy, participation, quality of life, sensory integration, sensory processing, well-being

## Abstract

**Background:**

Sensory processing allows us to interpret and respond adaptively to internal and external stimuli. Its alteration can cause difficulties in sensory modulation, integration or discrimination, affecting negatively occupational performance, functional participation, well-being, and quality of life. Understanding these alterations is essential for designing personalised interventions that promote autonomy and participation in daily life.

**Objectives:**

To analyse the relationship between sensory processing and performance.

**Methodology:**

A systematic review with PROSPERO ID number is CRD420251102155, was conducted following the PRISMA protocol. Electronic searches were performed in PubMed, Scopus, and Web of Science, including studies published between 2015 and 2025. Participants with or without a diagnosis of neurodevelopmental conditions from childhood to old age were considered, as well as interventions that analysed the relationship between sensory processing and occupational performance. Studies with a quantitative, qualitative or mixed approach were considered, with results reflecting the relationship between sensory processing and occupational performance assessed using a standardised or validated tool. Methodological quality was assessed using CASPe and STROBE, depending on the study design.

**Results:**

Twenty studies were included, with considerable heterogeneity in terms of design, sample, age, and variables. Consistent associations were identified between sensory processing disorders—especially hyperactivity, avoidance, and under-registration—and difficulties in personal autonomy, motor planning, social participation, emotional self-regulation, and psychological well-being.

**Conclusion:**

Sensory processing is key to understanding occupational performance. Sensory difficulties significantly affect occupational participation and individual functionality. Systematically assessing the sensory processing allows for more effective interventions. The importance of further research using rigorous methodologies, especially in undiagnosed adults and community settings, is highlighted.

**Systematic review registration:**

https://www.crd.york.ac.uk/PROSPERO/view/CRD420251102155; Registration number CRD42025110215.

## 1 Introduction

The Central Nervous System (CNS) is capable of receiving information from both the external environment and the body’s internal environment via the senses, processing it in an organised manner, and generating responses that enable us to adapt to each situation. This system of communication between the body and the brain is known as sensory processing (SP) (Del Moral Orro et al., 2013).

Sensory processing is divided into four phases: registration (individual identification of each stimulus), modulation/regulation (regulating the intensity with which we perceive the stimulus), discrimination (organising and interpreting stimuli, differentiating their characteristics and relevance) and integration (combining sensory information from various sources to interpret the environment and respond appropriately) (Del Moral Orro et al., 2013; Erazo-Santander, 2016; Díaz Benito and Yagüe, 2017; Grobelna et al., 2024; Pizarro et al., 2022; Lane et al., 2019).

Many people experience limitations in their daily lives, such as difficulty concentrating, discomfort with certain sounds or textures, motor clumsiness, or intense emotional reactions, without knowing that the origin may lie in a Sensory Processing Disorder (SPD). These alterations are not always obvious or diagnosed, which can lead to misinterpretations of behavior, both by those around them and by the person themselves. Occupational therapy, through the Sensory Integration approach, offers a specialised intervention method that allows these difficulties to be identified and addressed (Polatajko and Cantín, 2010; Williams, 2017).

Sensory processing involves the nervous system’s ability to receive, organise and interpret information received through the senses. When this process is disrupted, individuals may react disproportionately or inappropriately to everyday stimuli, affecting their performance in basic activities such as dressing, eating, working, studying or interacting with others (Erazo-Santander, 2016; Díaz Benito and Yagüe, 2017; Grobelna et al., 2024; Pizarro et al., 2022).

This process is essential for us to function effectively in our daily activities. Our brain constantly receives information from the senses and organises it to generate appropriate responses. This ability allows us to do everything from brushing our teeth without looking to maintaining our balance when walking or regulating our emotions in a noisy environment (Ayres, 1998; Blanche, 2008).

When it functions effectively, we adapt fluidly to environmental stimuli. However, if there is a dysfunction, difficulties may arise in areas such as self-care, academic performance, social participation, or even emotional regulation (Imperatore Blanche, 2005). Among the different possible manifestations, a person with tactile hypersensitivity may avoid wearing certain clothes, while another with hyporeactivity may constantly seek intense stimuli, affecting their concentration or behavior (Blanche, 2008; Imperatore Blanche, 2005).

Research in the field of sensory processing is essential, as it offers an opportunity to integrate knowledge about how differences in sensory processing can affect occupational performance. In recent years, it has gained increasing interest due to its significant impact on participation and performance in activities of daily living, both in neurotypical and neurodivergent populations (people with autism spectrum disorder, attention deficit hyperactivity disorder or dyslexia) (Barbarán Zumba, 2023; Beaudry, 2006).

Based on this, the objective of this review was to analyse the relationship between sensory processing and occupational performance through a systematic review of previous studies.

## 2 Materials and methods

### 2.1 Study design

This systematic review was conducted following the PRISMA statement (Hutton et al., 2016). The review protocol is available on PROSPERO with registration number CRD420251102155.

### 2.2 Search strategy

To identify relevant studies, a search was conducted in the following databases: PubMed, Scopus, Web of Science, and Cochrane. The terms used for the search were the following MeSH terms: ‘sensory processing’, ‘occupational therapy’, ‘activities of daily life’, “context,” and ‘quality of life’. All of them were separated by the Boolean operator AND and, in some search strategies, OR. The database search was conducted between October and May 2025.

### 2.3 Inclusion and exclusion criteria

The selection criteria were established following the PICO model (population, intervention, comparison, and outcomes).

The inclusion criteria were:

1. Population: Participants with or without a diagnosis of neurodevelopmental conditions from childhood to old age (age range: 2 to 100 years).
2. Intervention: Interventions that analysed the relationship between sensory processing and occupational performance.
3. Comparison: Studies not restricted to a single fixed comparison group.
4. Outcome measures: outcomes that reflect the relationship between sensory processing and occupational performance assessed with a standardised or validated tool.
5. Type of studies: Quantitative, qualitative, or mixed-method studies published in the last 10 years in English or Spanish.

The exclusion criteria established were:

- Studies that do not explicitly address the link between sensory processing and occupational performance.
- Studies with insufficient data for analysis.

### 2.4 Selection of studies

Based on the results obtained from the various search engines mentioned above, two independent researchers selected articles based on whether or not they met the inclusion criteria. In the event of disagreement, a third researcher intervened to make the final decision. First, articles were selected based on their titles when searching the various databases. Next, the abstracts were read to determine whether the selected studies were suitable for this review. The full studies were then read and critically analysed. Finally, studies that met the inclusion criteria were included in the review.

The following data were obtained from the studies included in the review: study design, study objective, sample characteristics, description of the intervention, study duration, outcome measures, and study results. These data were compiled in a standard table.

### 2.5 Methodological quality assessment

To ensure a rigorous and consistent analysis of the methodological quality of the studies included in this review, specific assessment tools were used according to the type of design of each study.

For cross-sectional comparative studies between groups (cases and controls), cohort studies, or intervention studies (clinical trials), the CASPe scale (Critical Appraisal Skills Programme in its Spanish version) (Prieto Botella and Company Devesa, 2023) was used, a widely used tool for the critical reading of scientific studies. It consists of specific checklists depending on the study design. This structure allows for systematic assessment of the methodological rigour of the study, the robustness of its results, and its relevance to professional practice. Its use facilitates the identification of biases, design errors, or limitations that may compromise the validity of the conclusions.

In the case of cross-sectional observational studies without a comparison group, the STROBE (Strengthening the Reporting of Observational Studies in Epidemiology) guidelines were used (Vandenbroucke et al., 2009). These consist of 22 items and are applied to improve the quality of observational study reports (cohort, case–control, and cross-sectional). It does not directly assess methodological quality, but rather the quality of the presentation and transparency of the study, which facilitates its critical interpretation.

## 3 Results

**Figure 1:**
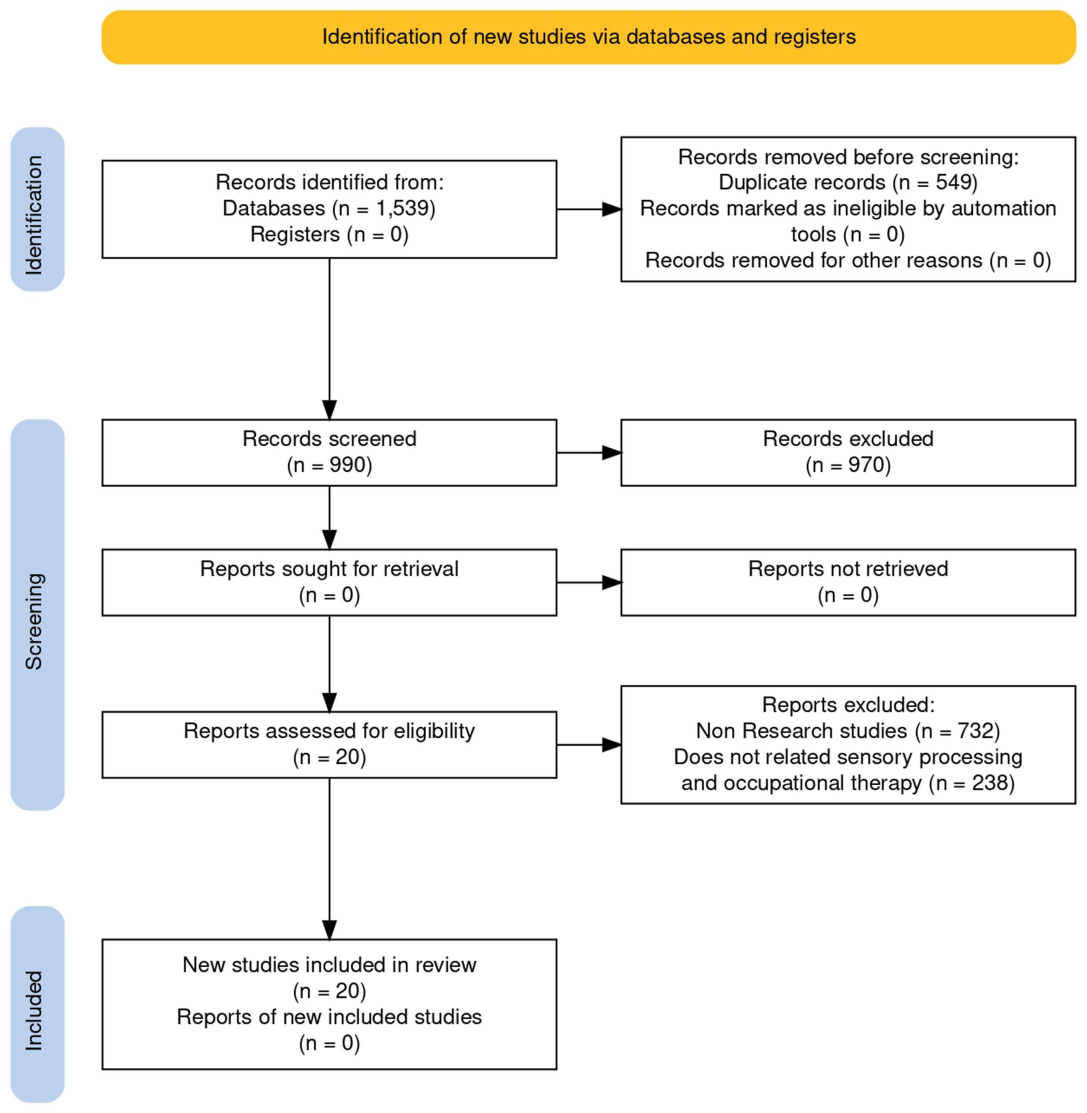
PRISMA flow diagram.

A total of 1,539 studies were obtained from the search of all databases. The PRISMA flow diagram (Figure 1) shows the search process and the study selection process. Duplicate records were excluded and analysed. Finally, 20 studies were included in the review.

From a methodological perspective, observational and comparative cross-sectional designs predominated. Longitudinal, quasi-experimental, and controlled clinical trials were also identified, providing a rich diversity of approaches. The main characteristics of the included studies are summarised in Table 1.

**Table 1:** Analysis of the studies reviewed.

Author	Type of study	Objectives	Sample	Intervention	Duration	Outcome measures and assessment tools	Results
Rajaei et al. (2020)	Descriptive cross-sectional study	To analyse the relationship between SP patterns and sleep quality in primary school children.	*N* = 231 Primary school pupils from Tehran. Aged between 7 and 12 years old. 133 female and 98 male.	Different questionnaires were distributed to all parent and the data was analysed statistically. Workshops were about students’ sleep problems were conducted and lasted approximately 3 hours.	Not specified.	Outcome measures: SP patterns (recording, searching, sensitivity, and avoidance) Assessment tools: Demographic information questionnaire. Children’s Sleep Habits Questionnaire (CSHQ). Sensory Profile Questionnaire (SPQ).	The relationship between SP patterns and sleep habit scores was statistically significant (*p* < 0.001). Each of the SP patterns was negatively related to areas of sleep habits (*p* = 0.005). There was a significant difference in SP (recording, searching, sensitivity, and avoidance) between children with greater difficulty maintaining sleep and children with normal sleep patterns (*p* < 0.001).
May-Benson et al. (2022)	Longitudinal observational follow-up study.	To explore the relationship between sensorimotor patterns in childhood and quality of life in adulthood and analyse the current relationship in adults with difficulties.	*N* = 53 Adults who received OT with ASI in childhood and were discharged before 2014, aged between 18 and 50 years old with no diagnoses other than sensorimotor problems.	The following data was collected: Childhood data (clinical records). Adult data: Online survey. Typical group: *N* = 26. Mild sensorimotor challenges group: N = 13. Defined sensorimotor challenges group: *N* = 14.	Not specified.	Outcome measures: Sensory-motor characteristics in childhood and adulthood; Quality of life in adulthood assessed in its four domains (physical health, psychological health, social relationships, and environment). Assessment tools: SXH WHOQOL-BREF ASH CENIZA.	In relation to adults with sensorimotor difficulties in childhood, there was a relationship between general sensorimotor functioning and physical health (*p* = 0.018), visual processing (*p* = 0.001), vestibular processing (*p* = 0.042) and tactile processing (*p* = 0.008). Visual processing was associated with psychological health (*p* = 0.024). Those who had typical functioning in adulthood showed a good quality of life.
Benarous et al. (2020)	Comparative cross-sectional observational study.	To determine the frequency of SPD in young people with DMDD compared to clinical and non-clinical controls; types of SPD in young people with DMDD and the relationship between SPD and emotional dysregulation.	*N* = 30 Patients with ADHD aged 6 to 16 years who attended a university hospital in outpatient or inpatient settings.	After the written informed consent, the study questionnaires were completed to proceed subsequently to the statistical analysis. Participants with a diagnosis of DMDD (*N* = 30). Participants without a diagnosis of DMDD (*N* = 18).	Not specified.	Outcome measures: Presence or absence of a diagnosis of ADHD. Presence and type of PDD. Degree of emotional dysregulation. Associated psychopathological dimensions (externalising behaviors, depressive symptoms, etc.). Assessment tools: Dunn Sensory Profile K-SADS-PL ALS-54 CBCL BDI-II.	The results indicated that young people with DMDD showed more SPD symptoms than those without (*p* = 0.405). The low register type was more prevalent in the DMDD group than in those with other emotional issues (*p* = 0.042), though no differences were observed for the other types.
Yim Loh et al. (2023)	Comparative cross-sectional observational study.	To examine PS and child participation in children with ASD (aged 6 to 10 years) and typical children of the same age and gender.	*N* = 186 Parents of Malaysian children aged 6 to 10 years diagnosed with ASD. Parents of Malaysian children aged 6 to 10 years with normal development. All parents must be able to speak and read Malay.	After receiving the information sheet and consent form, both questionnaires were completed. Case group: Parents of children with ASD. *N* = 93. Control group: Parents of “typical” children. *N* = 93.	Not specified.	Outcome measures: SP difficulties. Child participation (level of difficulty, frequency of participation, and level of enjoyment during participation). Assessment tools: Parental demographic questionnaire Child profile questionnaire SSP PICO.	Children with ASD showed lower participation (level, frequency, and enjoyment) than ‘typical’ children (p < 0.001). Differences in SP difficulties were significant across groups (*p* < 0.05) in all areas except motion sensitivity (*p* > 0.28). Auditory filtering showed significant correlations in difficulty (*p* < 0.01), frequency (*p* < 0.05) and enjoyment (*p* < 0.01), reducing participation among children in the ASD group.
Jovellar-Isiegas et al. (2020)	Comparative cross-sectional study.	To explore the differences in PS between children with PKU and children with TD and to analyse the relationship between SP and functional performance and quality of life.	*N* = 53 UCP group: Children aged 6–15 with UPC (no toxin 4 months prior) without cognitive impairment and attending school in the US. TD group: Children aged 6–15 with no history of neurological disorders, no SPD, no OT treatment and no physiotherapy.	Non-probability convenience sampling. Groups of cases recruited from AIDIMO and Hemiweb Control group, convenience sample. UPC group *N* = 29. TD group *N* = 24. The intervention consisted of standardized assessments (Sensory Profile 2, PEDI-CAT, Kidscreen-27/10).	Two different days: Day 1: a TO explains how to complete the CSP-2. Day 2: complete PEDI-CAT and Kidscreen.	Outcome measures: Child’s condition (UPC or DT) SP Functional performance Health-related quality of life. Assessment tools: CSP – 2 PEDI-CAT Kidscreen.	Children with PKU showed difficulties in SP (avoidance, registration, body position, oral, socio-emotional) and lower scores in functional performance (daily activities, mobility, social/cognitive and physical well-being).
Engel-Yeger and Kessel (2024)	Comparative cross-sectional observational study.	To specify activity preferences for children with atopic diseases and healthy children, and specify the SMDs in each atopic group and activity preference.	*N* = 253 Case group: Children aged 4 to 11 years with asthma. Children aged 4 to 11 years with AD. Children aged 4 to 11 years with rhinitis. Control group: Healthy children.	Meeting with parents to complete the SSP and their children the PAC. Case group: Children with asthma: *N* = 37 Children with AD: *N* = 37 Children with rhinitis: *N* = 31 Control group: *N* = 148	Not specified.	Outcome measures: Presence of atopic disease (asthma, atopic dermatitis, allergic rhinitis); Preference for daily activities; SMD; Relationship between SMD and participation according to atopic group. Assessment tools: PAC Sociodemographic questionnaire SSP.	Children with ADHD (girls) showed the greatest preference for activities, while children with asthma showed the least preference. Children with asthma preferred skill-based and informal activities. Atopic diseases can restrict participation in activities, leading to a preference for quiet activities.
Fernández-Andrés et al. (2018)	Comparative cross-sectional observational study.	To compare MS weaknesses in sensory modalities between children with ASD and typically developing children in the home and school environments.	N = 79 Case group: White children aged 5–8 years with ASD. Control group: White children aged 5–8 years with typical development.	After the children were recruited, interviews were conducted with their parents and teachers. Each child was assessed individually by the school counsellor. Case group: *N* = 41 Control group: *N* = 38	Three months. Data were collected from September to December 2013.	Outcome measures: Presence of ASD versus DT. Types of sensory modulation vulnerability (Hyper reactivity, Hypo reactivity, Sensory seeking). ASSESSMENT TOOLS: CPM GARS-2 SPM Sociodemographic questionnaires.	The ASD group obtained scores that showed higher levels of dysfunction in both environments: In the family environment the results showed a statistically significant under-response (*p* = 0.001). In the case of hyper-response and sensory seeking, no differences were found in some sensory modalities of the family environment hyper-response (*p* = < 0.001) and sensory seeking (*p* = < 0.001). Differences were found in some sensory modalities in the family environment. Hypersensitivity (*p* = < 0.001) and sensory seeking (*p* = < 0.001). In the school environment the results showed a statistically significant level of hyposensitivity (*p* = <0.001), hypersensitivity (*p* = < 0.001) and sensory seeking (*p* = < 0.001).
Kirby et al. (2019)	Longitudinal description.	To determine sensory associations of sensory response in children with ASD and other DD. Examine interactions by group and use of services.	*N* = 81 Children aged 2 to 12 with ASD. Children aged 2 to 12 with DD. Caregivers of the children included in the study.	Children participated in a comprehensive assessment. A primary caregiver reported on the services their child had used. Children with ASD: *N* = 50 Children with DD: *N* = 31 The data was collected at two time points (T1 and T2).	3.3 years.	Outcome measures: Early sensory characteristics; diagnosis (ASD or DD); use of services; family functioning. Assessment tools: SEQ; SP-2; SP Assessment for Young Children; Tactile Discrimination and Defensiveness Test; MSEL; -Stanford-Binet; -CGSQ; -HCAS.	The services were significant in relation to educational services (*p* < 0.05) and therapy services (*p* < 0.05). Sensory characteristics can affect the everyday experiences of both children and their carers.
González et al. (2021)	Comparative cross-sectional description.	To identify differences between Spanish children with ASD and neurotypical development in relation to ADL, happiness and SP.	*N* = 40 Children aged between 4 and 10 years old. ASD diagnosis without additional comorbid disorders. Informed consent signed by the father, mother or legal guardian.	Questionnaires and scales were administered to the children’s parents or carers during a personal interview lasting approximately 1 h and 15 min Children with ASD: *N* = 20. Neurotypical children: N = 20.	Not specified.	Outcome measures: Presence of ASD diagnosis or neurotypical development; Functional skills in ADLs; Level of enjoyment in play; Reactivity and SP. Assessment tools: PEDI; ToP; SPM; Sociodemographic questionnaire.	The results showed that the SP of children with ASD was associated with decreased performance in functional ADL skills and happiness compared to neurotypical children. Sensory reactivity problems were associated with poorer development in these occupational areas.
Newby et al. (2025)	Bidirectional cross-sectional observational study of cross-over cases.	To study the relationship between sensory reactivity and occupational performance in children with PANS during exacerbation phases.	*N* = 82 Children aged 5–13 years with PANS, without comorbid ASD or neuromotor disorders; PANS exacerbation within the previous 6 months; parents from English-speaking countries. Data collected at exacerbation and remission (two-period bidirectional crossover); only exacerbation data analyzed.	At the start of the study, participants were categorised as ET if they were in Exacerbation time or RT if they were in Remission time. The survey was updated monthly to determine whether they were ET or RT. If a child moved from ET to RT or vice versa, they were asked to complete a second round of the VABS and SPM surveys.	Data collection over 12 months.	Outcome measures: Sensory reactivity. Occupational performance (communication, daily living and socialisation skills). Gender, age, country of residence, use of therapeutic and educational services. Assessment tools: VABS; SPM; Demographic survey; Survey on the state of exacerbation.	The findings showed a significant relationship between sensory reactivity and occupational performance during PANS exacerbations, particularly in the areas of communication and daily living skills. Greater sensory reactivity is associated with decreased work performance during exacerbations.
Engel-Yeger and Rosenblum (2021)	Cross-sectional study.	To describe the prevalence of impaired PS in older adults and explore whether EF mediates between impaired SP and ADL performance in older adults.	*N* = 167 Healthy individuals aged 65 years and older. Adequate cognitive status. No symptoms of depression.	Home visit to sign informed consent and complete the various assessment tools.	Not specified.	Outcome measures: SP Executive functions. Performance in ADL. Assessment tools: MMSE; GDS; Sociodemographic and health questionnaire; AASP; BRIEF-A; DLQ.	The results showed that the participants had alteration in SP, mainly due to reduced ability to register and modulate sensory information from the everyday environment, in inhibition (*p* = 0.001), change efficiency (*p* = 0.004), working memory (*p* = 0.007), behavior regulation (*p* = 0.001), and GEC (*p* = 0.01). Executive dysfunction may exacerbate the negative impact of altered SP on older adults’ daily activities.
Lee et al. (2016)	Quasi-experimental trial with control group (non-randomised).	To investigate the impact of a SP intervention in the quality of life of students with sensory impairments.	*N* = 32 University students with problems in their SP ability.	SP was performed on the experimental group. Experimental group: *N* = 16. Control group: *N* = 16.	Six weeks.	Outcome Measures: SP. Quality of life. ASSESSMENT TOOLS: - AASP. WHO brief quality of life scale.	The experimental group showed a significant increase in all areas of quality of life (*p* = 0.001): Physical (*p* = 0.008). Psychological (*p* < 0.001). Social (*p* = 0.032). Environmental (*p* = 0.006). Using SP intervention methods in daily life can improve the quality of life for people with SP capacity difficulties.
Elbasan et al. (2022)	Comparative cross-sectional observational study.	To assess IS and ADL in children with TDC.	*N* = 72. Case group: Children aged 9 to 10 years with CDD. (16 girls and 21 boys) Control group: Children aged 9 to 10 years with normal development. (17 girls and 18 boys).	Case group: *N* = 37. The case group received a 12-week programme consisting of 12 sessions of 45 min each, with a frequency of 1 session per week. Control group: *N* = 35 The control group received a 12-week programme consisting of 12 sessions of 45 min each, with a frequency of 1 session per week.	12 weeks	Outcome measures: Presence of TDC versus DT; IS; Performance in ADL; Age, gender, and level of adaptive functioning. Asessment tools: Ayres Southern California Sensory Integration Test; WeeFIM.	Significant differences were found in the following areas: visual perception of shape and position in space; copying of designs; kinesthesia; manual perception of shape; finger identification; figure-ground perception; location of tactile stimuli; and perception of double tactile stimuli. The control group performed better in motor planning. Comprehension, expression, social communication, problem solving and memory were also significantly better in the control group (*p* < 0.05). There was a correlation between graphesthesia and self-care (*p* = 0.002) between the groups.
Pavãoa et al. (2021)	Cross-sectional observational study.	To investigate whether SPD is associated with activity performance in children with cerebral palsy and Gross Motor Function Classification System (GMFCS) levels I and II.	*N* = 28 (16 boys and 16 girls; with GMFCS I18 boys and II 10 boys). Children aged 5–15 years with CP and without ADHD or ASD participating in physical rehabilitation programmes, with lower limb muscle tone MMII 0 or 1 and ability to follow simple commands.	*N* = 28 Meeting with the primary caregiver to assess the children’s SP and sensory function.	Five days.	Outcome measures: SP. Functional activity performance. Caregiver assistance required. Assessment tools: SP; PEDI.	The results showed that SP in children with GMFCS levels I–II cerebral palsy (CP) was associated with their ability to perform activities of daily living and interact socially with their environment (*p* < 0.05).
Hochreuter et al. (2025)	Randomised trial (without clinical therapeutic intervention).	The aim of this study was to explore whether SPS is associated with pain threshold and tolerance in healthy adolescents, and to examine differences in pain perception following the induction of positive, negative or neutral mood states.	*N* = 100 Healthy adolescents aged 16 to 19 years old without chronic diseases affecting tactile or thermal modalities. No psychological or psychiatric treatment or medication affecting the perception of pain.	Questionnaires, heat pain exposure test, and initial assessment of pain threshold and tolerance. After that, they were asked to watch a series of 20 pictures from the International Affective Picture System (IAPS)0.40 Participants were randomly allocated to view positively (N = 34), negatively (N = 33), or neutrally (N = 33) valenced pictures.	Between June 2021 and April 2022.	Outcome measures: SP sensitivity. Pain tolerance, intensity, and threshold. Emotional regulation, parenting style, and social support. Assessment tools: HSC; ERQ; PBI; CASSS; IAPS.	The results of the study showed that, at the start of the study, highly sensitive adolescents had the lowest pain tolerance and threshold scores (*p* < 0.01). Significant differences were observed in initial pain threshold and tolerance scores between sensitivity groups (p < 0.01 and *p* < 0.005, respectively).
Ptak et al. (2022)	Comparative cross-sectional observational study.	To analyse whether there is a correlation between the onset of SPD and the timing or mode of delivery.	*N* = 75 Healthy children aged 6 to 9 years. No motor or intellectual disability. Not receiving psychiatric treatment.	Group I (*N* = 25): Children born before 37 weeks of gestation. Group II (*N* = 25): Children born by caesarean section between 38 and 40 weeks of gestation. Group III - control group (N = 25): Children born at term	January and February 2018.	Outcome measures: Type and timing of delivery. Presence of SPD. Assessment tools: Sensory-motor history questionnaire.	The results of the study showed that the timing and mode of delivery influence SPD. SPD was detected in 84% of premature babies and 80% of babies born by caesarean section, which is statistically significant. These groups are therefore at a higher risk of developing SPD than babies born at term by vaginal delivery.
Iimura and Takasugi (2022)	Cross-sectional observational study.	To examine the association between gastrointestinal symptoms as an indicator of physical health and SPS.	*N* = 863 (450 women and 413 men). Japanese adults aged 20 to 39.	Participants complete an online questionnaire. Sociodemographic characteristics are statistically controlled, and SPS was examined for correlation with gastrointestinal symptoms.	Not specified.	Outcome measures: SPS. Gastrointestinal symptoms. Sociodemographic factors. Assessment tools: HSP; Gastrointestinal Symptom Rating Scale; Demographic characteristics.	The results showed that high SPS is associated with physical health. Even when statistically controlling for participants’ sociodemographic characteristics, the data showed that highly sensitive people are more likely to experience a wide range of gastrointestinal symptoms, including reflux, abdominal pain, indigestion, diarrhoea and constipation,
Little et al. (2019)	Cross-sectional comparative design.	Assess PS among children with chronic constipation and without constipation, and specific SP patterns and modality scores on atypical toilet behavior.	*N* = 132 Children aged 3 to 5 years with constipation. Children aged 3 to 5 years without constipation. Children without ASD, severe developmental delay or neurological impairment.	Parents provided basic demographic information and information about their children’s toilet habits and sensory experiences Constipation group: N = 66. Control group: N = 66.	January 2015 and April 2017.	Outcome measures: SP patterns. Atypical toilet use behaviors. Assessment tools: SP-2; Toilet Habit Profile.	Children with chronic constipation showed significantly higher sensory scores than the control group, specifically in oral processing (*p* < 0.001), visual processing (*p* < 0.05), sensory avoidance (*p* < 0.001), and sensory sensitivity (*p* < 0.05). Children with chronic constipation have underlying sensory characteristics that contribute to difficulties with toileting.
Maldonado Gelder and Lugli Rivero (2020)	Comparative cross-sectional observational study.	To determine whether there is a difference in self-concept, self-efficacy, and quality of life between children with and without DPS.	*N* = 98 Children aged 7 to 13 years. Residents and schoolchildren in the city of Caracas. With and without a diagnosis of SP deficit.	The study involved applying the various rating scales to the sample Group with SP deficit: *N* = 38. Group without SP deficit: *N* = 60.	Not specified.	Outcome measures: Presence or absence of DPS. Self-concept. Self-efficacy. Quality of life. Assessment tools: SSP; Piers-Harris Children’s Self-Concept Assessment Scale; Children’s Self-Efficacy Scale; KIDSCREEN-52.	No significant differences were found in academic self-efficacy, although children with DPS showed lower mean scores. In terms of physical self-concept, lower scores were observed in children without deficits (*p* = 0.018). No significant differences were found in quality of life between the two groups.

*N*, sample; SP, Sensory Processing; PE, Primary Education; ASI, Ayres® Sensory Integration Therapy; SPD, Sensory Processing Difficulties; DMDD, Disruptive Mood Dysregulation Disorder; K-SADS-PL, Schedule for Affective Disorders and Schizophrenia for School-Age Children—Present and Lifetime version; ALS-54, Affective Lability Scale; CBCL, Child Behavior Checklist (4–18 years); BDI – II, Beck Depression Inventory (2nd edition); ASD, Autism Spectrum Disorder; SSP, Short Sensory Profile; PICO, Participation in Children’s Occupation; SIT, Sensory Integration Therapy; UC, Usual Care; OTs, Occupational Therapists; UCP, Unilateral Cerebral Palsy; TD, Typical Development; CSP -2, Child Sensory Profile; PEDI-CAT, Paediatric Evaluation of Disability Inventory; KIDSCREEN, Health-related quality of life in children; SE, Special Education; AIDIMO, Association for Research into Motor Disabilities; HEMIWEB, Association for Childhood Hemiparesis; SMD, Sensory Modulation Difficulties; AD, Atopic Dermatitis; HSP, Health Study Plan; PAC, Preference for Children’s Activities; ADD, Attention Deficit Disorder; MSD, Musculoskeletal Disorders; SM, Sensory Modulation; CPM: Colour progressive matrix of Raven’s Progressive Matrices Manual; GARS-2, Guilliam Autism Rating Scale; SPM, Sensory Processing Measure; SIRS, Sensory interests, repetitions, and seeking behaviors; DD, Other Developmental Disabilities; SEQ, Sensory Experiences Questionnaire; SP-2, Dunn Sensory Profile; MSEL; Mullen Early Learning Scale; CGSQ, Caregiver Stress Questionnaire; HCAS, Home and Community Activities Scale; ADL, Activities of Daily Living; PEDI, Paediatric Evaluation of Disability Inventory; ToP, Test of Playfulness; SPM, Sensory Processing Measure; PANS, Paediatric Acute-onset Neuropsychiatric Syndrome; VABS, Vineland Behavior Rating Scale; ET, Exacerbation time; RT, Remission time; EF, Executive Functions; MMSE, Mini Mental State Examination; GDS, Geriatric Depression Scale; AASP, Adolescent/Adult Sensory Profile; BRIEF-A, Behavior Rating Inventory of Executive Functioning, Adult Version; DLQ, Daily Living Questionnaire; CDD, Developmental Coordination Disorder; WeeFIM, Functional Independence Measure for Children; SPD, Sensory Processing Disorder; GMFCS, Gross Motor Function Classification System; SPS, Sensory Processing Sensitivity; HSC, Highly Sensitive Children Scale; ERQ, Emotion Regulation Questionnaire; PBI, Parental Bonding Instrument; CASSS, Social Support Scale for Children and Adolescents; IAPS, International Affective Picture System; HSP, Aesthetic Sensitivity Scale Japanese version.

### 3.1 Sociodemographic and methodological characteristics

In terms of sample size, the studies included showed wide variability, with samples ranging from 28 to 253 participants. The study by Pavãoa et al. (2021) had the smallest sample (*n* = 28), focusing on children with spastic cerebral palsy, while Engel-Yeger and Kessel (2024) recruited the largest number of participants (n = 253), including children with different atopic diseases.

The mean age of the participants was mainly in the 6–11 age range, i.e., childhood. The lowest age was observed in the study by Little et al. (2019), with children aged 3 to 5 years, while the highest was in the study by Engel-Yeger and Rosenblum (2021), which focused on older adults with an average age of 74.2 years.

This review covers a wide variety of clinical and non-clinical populations in which sensory processing disorders are studied. The most common pathologies included in the samples analysed were neurodevelopmental disorders, such as autism spectrum disorder (ASD), attention deficit hyperactivity disorder (ADHD), Down syndrome, fetal alcohol syndrome, developmental coordination disorder (DCD), disruptive mood dysregulation disorder (DMDD), and various forms of cerebral palsy (unilateral and mild to moderate spastic). In addition, emerging paediatric conditions such as paediatric acute-onset neuropsychiatric syndrome (PANS) and immunological diseases such as atopic diseases (atopic dermatitis, asthma and allergic rhinitis) were included in the analysed studies. On the other hand, samples of older adults, adolescents, and university students without clinical diagnosis but with dysfunctional sensory profiles were also included. Groups with specific conditions such as chronic functional constipation and high sensitivity to pain are also considered, analysing the impact of sensory profile on psychological well-being, functionality and quality of life. Overall, the studies show that sensory difficulties appear in both clinical and community contexts, reinforcing their relevance as a cross-sectional variable in the analysis of performance and participation.

From a methodological perspective, observational and comparative cross-sectional designs predominated (Benarous et al., 2020; Yim Loh et al., 2023; Randell et al., 2019; Jovellar-Isiegas et al., 2020; Engel-Yeger and Kessel, 2024; Fernández-Andrés et al., 2018; González et al., 2021; Newby et al., 2025; Engel-Yeger and Rosenblum, 2021; Elbasan et al., 2022; Pavãoa et al., 2021; Hochreuter et al., 2025; Ptak et al., 2022; Iimura and Takasugi, 2022; Little et al., 2019; Maldonado Gelder and Lugli Rivero, 2020). Longitudinal studies (May-Benson et al., 2022; Kirby et al., 2019), quasi-experimental studies (Lee et al., 2016), and controlled clinical trials (Randell et al., 2019) were also identified, providing a rich diversity of approaches.

With regard to the duration of the studies, cross-sectional designs with one-off data collection predominated. However, studies with a longer time frame were also included those conducted by Kirby et al. (2019) and Randell et al. (2019). The longitudinal study by Kirby et al. (2019) had an average follow-up of 3.3 years and the randomised clinical trial by Randell et al. (2019) carried out an intervention that lasted 26 weeks, with assessments at 6 and 12 months. The shortest study was that of Lee et al. (2016), with a sensory intervention lasting only 6 weeks.

The assessment tools used were diverse, but they maintained a certain theoretical consistency. Extensive use was identified of sensory profiles based on Dunn’s model, in its versions for children such as SP-2 (Sensory Profile 2) (Newby et al., 2025; Little et al., 2019; Maldonado Gelder and Lugli Rivero, 2020), SSP (Short Sensory Profile) (Yim Loh et al., 2023; Engel-Yeger and Kessel, 2024), CSP (Child Sensory Profile) (Jovellar-Isiegas et al., 2020; González et al., 2021), SPM (Sensory Processing Measure) (Fernández-Andrés et al., 2018; Newby et al., 2025) and adults AASP (Adolescent/Adult Sensory Profile) (Engel-Yeger and Rosenblum, 2021; Lee et al., 2016; Iimura and Takasugi, 2022). Quality of life was mainly measured using the WHOQOL-BREF (World Health Organization Quality of Life – BREF) (Engel-Yeger and Rosenblum, 2021; Lee et al., 2016), KIDSCREEN (Randell et al., 2019; Jovellar-Isiegas et al., 2020; Maldonado Gelder and Lugli Rivero, 2020) and PedsQL (Paediatric Quality of Life Inventory) (Jovellar-Isiegas et al., 2020; González et al., 2021; Elbasan et al., 2022). Other relevant tools included adaptive function scales such as VABS-3 (Vineland Adaptive Behavior Scales, Third Edition) (Randell et al., 2019; Newby et al., 2025) and PEDI (Paediatric Evaluation of Disability Inventory) (Jovellar-Isiegas et al., 2020; González et al., 2021; Pavãoa et al., 2021), Behavior Rating Inventory of Executive Function®) - Adult Version (Engel-Yeger and Rosenblum, 2021), Bandura self-efficacy (González et al., 2021), and specific measures of participation such as PAC (Preferences for Activities of Children) (Engel-Yeger and Kessel, 2024), PICO (Participation in Children’s Occupation) (Yim Loh et al., 2023) and HCAS (Home and Community Activities Scale) (Kirby et al., 2019).

The studies included in this review carried out a variety of interventions aimed at addressing sensory difficulties. These approaches were the following:

- Ayres Sensory Integration (ASI®) interventions (Rajaei et al., 2020; Yim Loh et al., 2023; Randell et al., 2019; Kirby et al., 2019; Pavãoa et al., 2021), particularly in children with autism spectrum disorder, developmental coordination disorder and cerebral palsy. These interventions seek to generate adaptive responses through sensorimotor play and show improvements in self-regulation, functional performance, and participation skills.
- Contextual adaptations and an ecological approach (May-Benson et al., 2022; Benarous et al., 2020; Jovellar-Isiegas et al., 2020; Fernández-Andrés et al., 2018; González et al., 2021; Ptak et al., 2022) that adjust the environment (home, classroom, or community) to the individual’s sensory needs. Strategies such as environmental modification, sensory-safe routines, and the principle of ‘fair challenge’ are used to facilitate inclusion and meaningful participation.
- Interventions focused on the family and carers (Kirby et al., 2019; Newby et al., 2025) emphasised the importance of actively involving carers in understanding the child’s sensory profile and designing everyday activities. This has proven benefits for both the child’s performance and the reduction of family stress.
- Group programmes and sensory enrichment (Engel-Yeger and Kessel, 2024; Engel-Yeger and Rosenblum, 2021) that promote socialisation, movement and sensory self-regulation. These were implemented in both schools and occupational therapy centres with positive results in terms of participation and psychological well-being.
- Individualised interventions for adolescents and adults without a clinical diagnosis, using short, tailored sessions that produced significant improvements in quality of life in the physical, psychological, social and environmental domains (Lee et al., 2016).

Psychoeducational and emotional components were also included in the interventions, whereby sensory interventions were complemented by strategies that reinforce a positive perception of a child’s abilities, particularly in academic and social contexts (Maldonado Gelder and Lugli Rivero, 2020).

### 3.2 Results related to the variables and outcomes of the study

The most analysed variables were sensory processing (in its different profiles and modalities), occupational participation (Yim Loh et al., 2023; Randell et al., 2019; Jovellar-Isiegas et al., 2020; Engel-Yeger and Kessel, 2024; Fernández-Andrés et al., 2018; Kirby et al., 2019; González et al., 2021; Newby et al., 2025; Lee et al., 2016; Elbasan et al., 2022; Little et al., 2019), functional performance (Rajaei et al., 2020; Yim Loh et al., 2023; Randell et al., 2019; Jovellar-Isiegas et al., 2020; Kirby et al., 2019; González et al., 2021; Newby et al., 2025; Engel-Yeger and Rosenblum, 2021; Pavãoa et al., 2021; Little et al., 2019), quality of life (Yim Loh et al., 2023; Randell et al., 2019; Jovellar-Isiegas et al., 2020; Engel-Yeger and Kessel, 2024; González et al., 2021; Newby et al., 2025; Engel-Yeger and Rosenblum, 2021; Lee et al., 2016; Hochreuter et al., 2025; Maldonado Gelder and Lugli Rivero, 2020) and emotional or cognitive self-regulation (Benarous et al., 2020; Randell et al., 2019; Engel-Yeger and Kessel, 2024; Kirby et al., 2019; Engel-Yeger and Rosenblum, 2021; Hochreuter et al., 2025; Maldonado Gelder and Lugli Rivero, 2020). Several studies incorporated less frequent dimensions, such as pain perception (Hochreuter et al., 2025), family functioning (Kirby et al., 2019), gastrointestinal symptoms (Iimura and Takasugi, 2022) or self-concept (Maldonado Gelder and Lugli Rivero, 2020).

In addition to these general patterns, it was possible to identify more precisely the strength and direction of the associations described in the included studies. Most cross-sectional studies showed moderate or strong associations, generally of a negative nature, indicating that higher levels of hyperreactivity, hyporeactivity, avoidance or low registration were consistently associated with poorer functional performance, more limited participation, poorer emotional self-regulation and lower psychological well-being (Rajaei et al., 2020; May-Benson et al., 2022; Yim Loh et al., 2023; Jovellar-Isiegas et al., 2020; Engel-Yeger and Kessel, 2024; González et al., 2021; Newby et al., 2025; Engel-Yeger and Rosenblum, 2021). For example, Rajaei et al. (2020) found significant correlations between the four sensory patterns and sleep habits (*p* < 0.001), whilst Engel-Yeger and Rosenblum (2021) observed that sensory disturbances in older adults were associated with poorer executive functioning (*p* = 0.001–0.007) and poorer performance in activities of daily living.

Clear differences were also identified between age groups and between clinical and non-clinical populations. Children with autism spectrum disorder showed the highest levels of hyper-reactivity, hypo-reactivity and sensory seeking, alongside lower engagement and enjoyment in everyday activities (Yim Loh et al., 2023; Fernández-Andrés et al., 2018; González et al., 2021). Children with phenylketonuria presented difficulties with registration, avoidance and oral processing, associated with lower functional performance (Jovellar-Isiegas et al., 2020). In atopic conditions, children showed reduced participation and a preference for low-sensory-demand activities (Engel-Yeger and Kessel, 2024). In paediatric acute-onset neuropsychiatric syndrome (PANS), fluctuations in sensory reactivity during exacerbation phases were directly linked to poorer performance in communication and activities of daily living (Newby et al., 2025). In adults, sensorimotor functioning in childhood predicted quality of life in adulthood (May-Benson et al., 2022), whilst in older adults, sensory impairments were associated with poorer performance in activities of daily living and impaired executive functions (Engel-Yeger and Rosenblum, 2021).

With regard to intervention studies, only one randomised clinical trial met the inclusion criteria (Randell et al., 2019). This study showed that the intervention based on Ayres Sensory Integration^®^ produced significant improvements in irritability, agitation, adaptive skills and participation, with effects persisting at 6 and 12 months. Furthermore, a reduction in caregiver stress and an improvement in their quality of life were observed. This study provides robust evidence regarding the efficacy of interventions based on sensory integration in children with ASD.

In terms of the results, there were significant points of consistency between the studies. In general, sensory processing difficulties, especially sensory avoidance profiles (Engel-Yeger and Kessel, 2024; Kirby et al., 2019; Newby et al., 2025; Little et al., 2019) were consistently associated with lower functional performance, limited participation, poorer self-regulation, and, in some cases, a greater presence of somatic or emotional symptoms.

This association was evident both in children with neurodevelopmental conditions (Rajaei et al., 2020; Benarous et al., 2020; Yim Loh et al., 2023; González et al., 2021; Newby et al., 2025; Pavãoa et al., 2021) and in healthy adults with high sensory sensitivity (Engel-Yeger and Rosenblum, 2021; Hochreuter et al., 2025; Iimura and Takasugi, 2022). Several studies (Engel-Yeger and Rosenblum, 2021; Lee et al., 2016) suggested that executive functions or the perception of self-efficacy mediate this relationship, while others (Randell et al., 2019; Kirby et al., 2019) showed that these difficulties also have an impact on carers and family contexts.

### 3.3 Methodological quality of the included studies

The results of the analysis of the methodological quality of the 20 articles included in this review using the CASPe and STROBE scales are shown in Tables 2–5 of the supplementary material.

**Table 2:** CASPe methodological scale for cohort studies.

CASPe: Cohort studies		
Item	May-Benson et al. (2022)	Kirby et al. (2019)
1. Does the study focus on a clearly defined topic?	Yes	Yes
2. Were the comparison groups recruited in an acceptable manner*?*	Yes	Yes
3. Was exposure measured acceptably to minimise bias?	Yes	Yes
4. Have confounding factors been identified and taken into account?	Partially	Partially
5. Were the groups comparable in all important respects except exposure?	Partially	Partially
6. Were the outcomes measured objectively to minimise bias?	Yes	Yes
7. Was the follow-up long enough?	Yes	Yes
8. Was the effect of incomplete follow-up taken into account?	Partially	Partially
9. What were the study’s results?	Yes	Yes
10. How accurate are the results?	Partially	Partially
11. Do you think the results are credible?	Yes	Yes
Estimated methodological quality	Very good	Very good

**Table 3:** CASPe methodological scale for case–control studies.

CASPe: cases and controls							
Item	Benarous et al. (2020)	Yim Loh et al. (2023)	Jovellar-Isiegas et al. (2020)	Engel-Yeger and Kessel (2024)	Fernández-Andrés et al. (2018)	González et al. (2021)	Elbasan et al. (2022)
Does the study focus on a clearly defined topic?	Yes	Yes	Yes	Yes	Yes	Yes	Yes
Have the authors used an appropriate method to answer the question?	Yes	Yes	Yes	Yes	Yes	Yes	Yes
Were the cases recruited/included in an acceptable manner?	Yes	Yes	Yes	Yes	Yes	Yes	Yes
Were the controls selected in an acceptable manner?	Yes	Yes	Yes	Yes	Yes	Yes	Yes
Was exposure measured objectively to minimise bias?	Yes	Yes	Yes	Yes	Yes	Yes	Yes
Were all important confounding factors identified?	Partially	Partially	Partially	Partially	Partially	Partially	Partially
Was the effect of confounding factors taken into account in the study design and/or analysis?	No	No	No	No	No	No	No
What results were obtained in the study?	Yes	Yes	Yes	Yes	Yes	Yes	Yes
How accurate are the results?	Partially	Partially	Partially	Partially	Partially	Partially	Partially
Do you think the results are credible?	Yes	Yes	Yes	Yes	Yes	Yes	Yes
Can the results be applied to your environment?	Yes	Yes	Yes	Yes	Yes	Yes	Yes
Estimated methodological quality	Good	Good	Good	Good	Good	Good	Good

**Table 4:** CASPe methodological scale for randomised controlled trials.

CASPe: randomised controlled trials		
Item	Randell et al. (2019)	Lee et al. (2016)
1. Was a research question clearly stated?	Yes	Yes
2. Was the sneed for the trial justified?	Yes	Yes
3. Was this randomised controlled trial appropriate for answering the question?	Yes	Yes
4. Were participants randomly assigned to groups?	Yes	Yes
5. Was the allocation adequately concealed?	Partially	No
6. Were the groups adequately compared at the start of the study?	Yes	Yes
7. Did all participants receive the treatment to which they were assigned?	Partially	Partially
8. Were all patients in the groups to which they were randomised analysed together?	Yes	Yes
9. Were the study outcomes measured objectively?	Yes	Yes
10. Are the study results credible?	Yes	Yes
11. How applicable are the study results to your work?	Yes	Partially
Estimated methodological quality	Very Good	Good

**Table 5:** STROBE methodological scale.

Strobe									
Items	Rajaei et al. (2020)	Newby et al. (2025)	Engel-Yeger and Rosenblum (2021)	Pavãoa et al. (2021)	Hochreuter et al. (2025)	Ptak et al. (2022)	Iimura and Takasugi (2022)	Little et al. (2019)	Maldonado Gelder and Lugli Rivero (2020)
1a. Indicate the study design with a commonly used term in the title or abstract.	Yes	Yes	Yes	Yes	Yes	Yes	Yes	Yes	Yes
1b. Provide an informative and balanced summary of what was done and found in the abstract.	Yes	Yes	Yes	Yes	Yes	Yes	Yes	Yes	Yes
2. Explain the scientific context and rationale for the study being reported	Yes	Yes	Yes	Yes	Yes	Yes	Yes	Yes	Yes
3. State the specific objectives, including any specified prior hypotheses.	Yes	Yes	Yes	Yes	Yes	Yes	Yes	Yes	Yes
4. Present the key elements of the study design at the beginning of the paper.	Yes	Yes	Yes	Yes	Yes	Yes	Yes	Yes	Yes
5. Describe the setting, locations, and relevant dates, including periods of recruitment, exposure, follow-up, and data collection	Yes	Yes	Yes	Yes	Yes	Yes	Yes	Yes	Yes
6a. Give the eligibility criteria and sources and methods of participant selection.	Yes	Yes	Yes	Yes	Yes	Yes	Yes	Yes	Yes
6b. Describe follow-up methods, if applicable.	N/a	N/a	N/a	N/a	N/a	N/a	N/a	N/a	N/a
7. Clearly define all outcomes, exposures, predictors, potential confounders, and effect modifiers.	Yes	Yes	Yes	Yes	Yes	Yes	Yes	Yes	Yes
8. For each variable of interest, provide data sources and details of assessment (measurement) methods.	Yes	Yes	Yes	Yes	Yes	Yes	Yes	Yes	Yes
9. Explain how the sample size was determined.	No	No	Yes	No	Yes	No	Yes	No	No
10. Explain how missing data were handled.	No	No	Partially	No	No	No	Yes	No	No
11a. Describe all statistical methods, including those used to control for confounding.	Partially	Partially	Partially	Partially	Partially	Partially	Yes	Partially	Partially
11b. Describe the methods used to examine subgroups and interactions.	No	No	Partially	No	Partially	No	No	No	No
11c. Explain how any missing data were handled.	No	No	No	N/a	No	No	Yes	No	No
11d. If sensitivity analyses were performed, describe them.	No	No	No	No	No	No	No	No	No
12a. Report the number of individuals at each stage of the study.	Yes	Yes	Yes	Yes	Yes	Yes	Yes	Yes	Yes
12b. Give reasons why participants were not included.	Yes	Yes	Yes	Yes	Yes	Yes	Yes	Partially	Partially
12c. Consider using a flow chart.	No	No	No	No	No	No	Yes	No	No
13a. Give characteristics of participants relevant to the study.	Yes	Yes	Yes	Yes	Yes	Yes	Yes	Yes	Yes
13b. Indicate the number of participants with missing data for each variable.	No	No	No	No	No	No	Yes	No	No
14a. Present unadjusted and, if relevant, adjusted estimates with their precision (95% confidence intervals).	No	No	Partially	Partially	Partially	Partially	Yes	Partially	No
14b. Report the categories if continuous outcome measures have been categorised.	Yes	Yes	Yes	Yes	Yes	Yes	Yes	Yes	Yes
14c. If applicable, translate relative risk estimates to absolute risk measures.	N/a	N/a	N/a	N/a	N/a	N/a	N/a	N/a	N/a
15. Report other analyses performed (e.g., subgroup analyses, interactions, or sensitivity).	No	No	Partially	No	Partially	No	No	Yes	No
16. Summarise key findings with respect to the study objectives.	Yes	Yes	Yes	Yes	Yes	Yes	Yes	Yes	Yes
17. Discuss limitations, including possible sources of bias or imprecision.	Yes	Yes	Yes	Yes	Yes	Yes	Yes	Yes	Yes
18. Provide an overall interpretation of the results considering other relevant studies.	Yes	Yes	Yes	Yes	Yes	Yes	Yes	Yes	Yes
19. Comment on the generalisability (external validity) of the study results.	No	Partially	Partially	Partially	Partially	Partially	Partially	Partially	Partially
20. Indicate the source of funding and the role of funders in the present study.	Yes	Yes	Yes	Yes	Yes	Yes	Yes	Yes	Yes
21. State approval by an ethics committee and informed consent, if applicable.	Yes	Yes	Yes	Yes	Yes	Yes	Yes	Yes	Yes
22. Provide information on how the study data and materials can be accessed, if applicable.	No	No	Partially	No	Partially	Partially	Partially	No	No
Estimated methodological quality	Good	Good	Very good	Good	Very good	Good	Very good	Good	Good

The methodological quality assessment showed considerable variability in the rigour of the studies. Based on the CASPe scoring criteria, the cohort studies (May-Benson et al., 2022; Kirby et al., 2019) presented high overall quality, with affirmative responses in most items. However, four aspects were rated as ‘Partial’ in both studies. Even so, the studies maintained adequate validity in the measurement of exposure and outcomes. They also had sufficient follow-up and an appropriate statistical analysis. This allows their conclusions to be considered relevant. However, there is a need for caution when considering these conclusions due to the methodological limitations detected.

The case–control studies (Benarous et al., 2020; Yim Loh et al., 2023; Jovellar-Isiegas et al., 2020; Engel-Yeger and Kessel, 2024; Fernández-Andrés et al., 2018; González et al., 2021; Elbasan et al., 2022) showed a consistent pattern of quality. In all of them, affirmative responses were obtained for most items, indicating adequate selection of cases and controls, valid measurement of exposure, and correct identification of confounding factors. However, two items were rated as ‘Partial’: equivalence in exposure measurement between cases and controls and consideration of the effect of losses or incomplete data. Besides, with regard to control of recall bias, all studies were rated as ‘No,’ which constitutes the main methodological limitation of the set. Despite these weaknesses, the studies presented adequate precision in their results and good clinical applicability, allowing their evidence to be considered relevant. However, However, this consideration should be treated with caution due to the potential biases identified.

The methodological assessment of the two randomised clinical trials (Randell et al., 2019; Lee et al., 2016) showed high overall quality. Both studies had adequate randomisation, sequence concealment, baseline similarity of groups, precision of results, and complete follow-up. However, common limitations were identified in the homogeneous measurement of outcomes. In addition, methodological differences were observed between the studies regarding the maintenance of comparability between groups and the applicability of the results. These differences suggest variations in the trials’ internal and external robustness, although both provided relevant evidence, bearing in mind the limitations identified.

The methodological quality of the nine observational studies assessed with the STROBE guideline showed a heterogeneous pattern of quality in relation to the description of the design, the definition of variables, the data sources, the descriptive results, and the final interpretation. All studies received a positive rating, indicating adequate transparency in the presentation of basic information. However, relevant limitations were identified in several key aspects. Bias control, sample size, handling of missing data, statistical methods for controlling confounding, subgroup analyses and adjusted estimates showed considerable variability between studies, with ‘No’ or “Partial” ratings predominating. Besides, sensitivity analysis was rated ‘No’ in all cases, which is one of the main methodological weaknesses of the set.

Differences were also observed in relation to the presentation of participant characteristics, the primary outcomes and transparency regarding data availability, with most studies failing to fully comply with STROBE criteria.

Despite these limitations, the studies showed consistent strengths in the description of the design, the clarity of the objectives, the definition of variables, and the interpretation of the findings, allowing their contribution to be considered relevant However, this results need to be considered with caution due to the methodological shortcomings detected.

## 4 Discussion

Sensory integration is an important neurological process through which the central nervous system organises and interprets stimuli from both the environment and the body itself (Del Moral Orro et al., 2013; Erazo-Santander, 2016; Díaz Benito and Yagüe, 2017; Ayres, 1998). When disruptions occur in this processing, the interpretation of sensory information may be altered or incomplete, leading to responses that do not match the demands of the stimulus. These patterns of sensory processing can influence arousal levels, attention span and behavioral regulation, thereby affecting performance in activities of daily living.

The aim of this study was to analyse the relationship between sensory processing and occupational performance through a systematic review of previous studies.

The results of this review show a high degree of consistency among the studies included with regard to the association between atypical sensory processing profiles, especially hyporeactivity, under-registration and avoidance, and a negative impact on daily functioning and participation (Rajaei et al., 2020; May-Benson et al., 2022; Benarous et al., 2020; Yim Loh et al., 2023; Randell et al., 2019; Jovellar-Isiegas et al., 2020; Engel-Yeger and Kessel, 2024; Fernández-Andrés et al., 2018; Kirby et al., 2019; González et al., 2021; Newby et al., 2025; Engel-Yeger and Rosenblum, 2021; Lee et al., 2016; Elbasan et al., 2022; Pavãoa et al., 2021; Hochreuter et al., 2025; Ptak et al., 2022; Iimura and Takasugi, 2022; Little et al., 2019; Maldonado Gelder and Lugli Rivero, 2020). This trend is consistent with previous literature, which has also documented the influence of these sensory patterns on self-efficacy, emotional regulation, and subjective well-being.

As suggested by several studies included in this review (Yim Loh et al., 2023; Randell et al., 2019; Fernández-Andrés et al., 2018; Kirby et al., 2019; Newby et al., 2025), sensory hyporeactivity appears to be one of the most consistent predictors of low participation in structured, social, and family activities, which coincides with findings in populations with autism spectrum disorder, developmental coordination disorder, and developmental disability. This trend is also observed in non-clinical contexts (Lee et al., 2016), suggesting that certain sensory patterns, even if they do not fit into a diagnosis, can have a significant functional impact in everyday life.

With regard to quality of life, the studies analysed indicate that sensory impairments can influence both physical and emotional aspects of the health-related quality of life (Jovellar-Isiegas et al., 2020; González et al., 2021; Hochreuter et al., 2025). However, Maldonado Gelder and Lugli Rivero (2020) found no significant differences between clinical groups and controls in this dimension. This inconsistency can be interpreted from several methodological and contextual angles, such as differences in participant characteristics (age and stage of development, symptom profile and sociocultural context), variability in assessment tools (different quality of life instruments and different sensory processing measures), influence of protective factors (high family support, early therapeutic intervention and inclusive educational environments) and differences in design and analysis (insufficient sample size, control of covariates and cross-sectional designs).

In terms of functional performance, studies on cerebral palsy (Jovellar-Isiegas et al., 2020; Pavãoa et al., 2021), acute-onset neuropsychiatric syndrome (Newby et al., 2025), developmental coordination disorder (Elbasan et al., 2022), and ageing (Engel-Yeger and Rosenblum, 2021) confirmed that the presence of deficits in sensory modulation and organisation directly impact independence in activities of daily living, mobility, social function, and efficiency in everyday tasks. This pattern is particularly enhanced when executive dysfunctions (Engel-Yeger and Rosenblum, 2021) or a lack of adaptive strategies are present. The observed relationship between sensory alterations and functional performance is consistent with the theoretical framework of sensory integration, which holds that participation in activities of daily living depends on the nervous system’s ability to modulate and discriminate between tactile, proprioceptive, vestibular, and visual information (Del Moral Orro et al., 2013; Díaz Benito and Yagüe, 2017; Ayres, 1998). Activities of daily living are inherently sensory activities, as they require precise discrimination, postural adjustment, motor planning, and emotional regulation. In relation to this, it is interesting to highlight that previous studies have demonstrated the effectiveness of sensory integration in developing fundamental learning skills in everyday activities that would later influence future occupational performance (Gladys Amparo and Gallegos Navas, 2020).

The influence of sensory processing on emotional and cognitive self-regulation is one of the least explored aspects. Although there is evidence of the close relation between sensation and emotion, various authors emphasise that the role of sensory processing as a regulatory mechanism remains an underdeveloped area that is poorly integrated into current models of self-regulation (Rodriguez and Kross, 2023). However, in this review, we found four studies showing that sensory difficulties correlate with low self-efficacy, greater parental stress, and lower emotional resfgulation, especially in children with autism spectrum disorder, atopic diseases, and non-specific sensory processing deficits (Randell et al., 2019; Engel-Yeger and Kessel, 2024; Kirby et al., 2019; Maldonado Gelder and Lugli Rivero, 2020). These findings support the need to include self-regulation strategies in the therapeutic approach, even when no explicit behavioral impairment is observed.

Finally, it should be noted that only four of the studies included in this review implemented structured sensory integration interventions (Randell et al., 2019; González et al., 2021; Lee et al., 2016; Elbasan et al., 2022), despite the fact that most of the studies showed significant sensory alterations but use very diverse approaches (psychomotor programmes, perceptual training, behavioral interventions or structured activities) that are not explicitly based on Ayres’ model. This methodological heterogeneity makes it difficult to compare studies and conditions the global and comprehensive interpretation of the results.

In the articles that did apply sensory integration (Randell et al., 2019; González et al., 2021; Lee et al., 2016; Elbasan et al., 2022), improvements were described in sensory modulation, behavioral organisation and occupational participation. These improvements were often related to the individualised, playful and participation-oriented nature of sensory integration, as well as its emphasis on active and meaningful sensorimotor experiences. In contrast, studies using interventions not based on sensory integration showed more variability in their results. Although some studies showed improvements in specific skills (such as coordination, attention, or self-regulation), these changes had a tendency to be partial and less generalisable to daily life (Jovellar-Isiegas et al., 2020; Engel-Yeger and Kessel, 2024; Kirby et al., 2019; Newby et al., 2025; Engel-Yeger and Rosenblum, 2021). The reason for this could be because such interventions focus on isolated components of performance rather than on the overall integration of sensory systems. This gap between diagnosis and action highlights the need to translate evidence into specific and reproducible clinical practices. The intervention conducted in university students (Lee et al., 2016) also suggested the applicability of sensory approaches in non-clinical populations, opening the door to promotion and prevention models from an occupational health perspective.

### 4.1 Limitations of the study

Even though the same selection criteria were applied, the wide range of designs, populations, and assessment tools meant that direct comparisons or generalisable conclusions could not be drawn.

Although the search strategy did not include terms related to neurodevelopmental disorders, many of the studies identified did focus on these populations. This means that sensory processing difficulties appear to be primarily associated with this group, limiting the generalisability of the findings to other populations. The majority of the evidence reviewed were studies with small sample sizes, no control groups or limitations in longitudinal follow-up, which weakens the level of scientific evidence available on interventions focusing on sensory processing. We consider that this circumstance could condition the strength of the clinical recommendations derived from the analysis.

Furthermore, most of the studies analysed in this review had observational, cross-sectional or correlational designs, which limits the possibility of establishing causal relationships between sensory profiles and functional, emotional or participatory outcomes.

Although many studies describe sensory difficulties in diagnosed populations, not all included active therapeutic implementations. This suggest that there is a gap between the sensory profile assessment and its translation into effective interventions, especially in non-specialised school, community or clinical settings.

These limitations should be considered when interpreting the results and underscore the need to continue developing robust, high-quality research in the field of sensory processing and its relationship to occupational performance.

## 5 Conclusion

- Sensory processing had a direct influence on autonomy, quality of life, and occupational participation, especially in people with neurodevelopmental disorders. Its assessment is key to guiding person-centred interventions.
- Sensory imparments, particularly hyporeactivity, low registration, and avoidance, are consistently associated with emotional dysregulation, functional difficulties, and limitations in daily life.
- Sensory difficulties significantly affect occupational participation and individual functionality, especially when they manifest themselves in environments with high sensory or social demands. These difficulties can limit a person’s ability to initiate, maintain, or complete everyday activities, impacting their autonomy, productivity, and sense of belonging.

## Data Availability

The original contributions presented in the study are included in the article/supplementary material, further inquiries can be directed to the corresponding author.
